# Fossilized Biophotonic Nanostructures Reveal the Original Colors of 47-Million-Year-Old Moths

**DOI:** 10.1371/journal.pbio.1001200

**Published:** 2011-11-15

**Authors:** Maria E. McNamara, Derek E. G. Briggs, Patrick J. Orr, Sonja Wedmann, Heeso Noh, Hui Cao

**Affiliations:** 1 Department of Geology & Geophysics, Yale University, New Haven, Connecticut, United States of America; 2 UCD School of Geological Sciences, University College Dublin, Belfield, Dublin, Ireland; 3 Yale Peabody Museum of Natural History, Yale University, New Haven, Connecticut, United States of America; 4 Senckenberg Forschungsinstitut und Naturmuseum, Forschungsstation Grube Messel, Messel, Germany; 5 Department of Applied Physics, Yale University, New Haven, Connecticut, United States of America; University of Bristol, United Kingdom

## Abstract

Structural colors are generated by scattering of light by variations in tissue nanostructure. They are widespread among animals and have been studied most extensively in butterflies and moths (Lepidoptera), which exhibit the widest diversity of photonic nanostructures, resultant colors, and visual effects of any extant organism. The evolution of structural coloration in lepidopterans, however, is poorly understood. Existing hypotheses based on phylogenetic and/or structural data are controversial and do not incorporate data from fossils. Here we report the first example of structurally colored scales in fossil lepidopterans; specimens are from the 47-million-year-old Messel oil shale (Germany). The preserved colors are generated by a multilayer reflector comprised of a stack of perforated laminae in the scale lumen; differently colored scales differ in their ultrastructure. The original colors were altered during fossilization but are reconstructed based upon preserved ultrastructural detail. The dorsal surface of the forewings was a yellow-green color that probably served as a dual-purpose defensive signal, i.e. aposematic during feeding and cryptic at rest. This visual signal was enhanced by suppression of iridescence (change in hue with viewing angle) achieved via two separate optical mechanisms: extensive perforation, and concave distortion, of the multilayer reflector. The fossils provide the first evidence, to our knowledge, for the function of structural color in fossils and demonstrate the feasibility of reconstructing color in non-metallic lepidopteran fossils. Plastic scale developmental processes and complex optical mechanisms for interspecific signaling had clearly evolved in lepidopterans by the mid-Eocene.

## Abstract 2

Original structural colors reconstructed in fossil moths had a dual defensive function and illuminate the evolution of communication strategies in insects.

## Author Summary

Biological structural colors are generated when light is scattered by nanostructures in tissues. Such colors have diverse functions for communication both among and between species. Structural colors are most complex in extant butterflies and moths (lepidopterans), but the evolution of such colors and their functions in this group of organisms is poorly understood. Fossils can provide insights into the evolution of biological structures, but evidence of structurally colored tissues was hitherto unknown in fossil lepidopterans. Here, we report the preservation of structurally colored scales in fossil moths with striking metallic hues from the ∼47-million-year-old (Eocene) GrubeMessel oil shales (Germany). We identify the color-producing nanostructure in the scales and show that the original colors were altered during fossilization. Preserved details in the scales allow us to reconstruct the original colors and show that the dorsal surface of the forewings was yellow-green. The optical properties of the scales strongly indicate that the color functioned as a warning signal during feeding but was cryptic when the moths were at rest. Our results confirm that structural colors can be reconstructed even in non-metallic lepidopteran fossils and show that defensive structural coloration had evolved in insects by the mid-Eocene.

## Introduction

Structural color has long been of interest to biologists. It is phenotypically significant in many organisms [1], forms the basis of diverse inter- and intra-specific communication strategies [2], and is implicated in pivotal evolutionary transitions [3]. Evidence of structural color has been reported from some fossil biotas [3]–[6], but has received little attention. This limits our ability to reconstruct the origins of activity patterns, habitat preferences, and social and sexual signaling mechanisms [7]. This is particularly problematic in the case of Lepidoptera (butterflies and moths), which exhibit the most complex and diverse structural colors of any living group of organisms [8]. Structural colors in extant lepidopterans are generated by modification of one or more components of the basic scale architecture (longitudinal ridges and transverse crossribs upon a basal lamella that is supported by columnar trabeculae in the scale lumen) into a biophotonic nanostructure of chitin and air [9]. Such color-generating multilayer structures can arise via specialization of the ridges and their ridge-lamellae, crossribs, or the scale lumen; the lumen can also exhibit various other modifications, including complex three-dimensional photonic crystals. The various color-producing nanostructures in lepidopteran scales may be related developmentally [9] and all generate color via interference of scattered light [1], although the overall visual effect can be influenced by other optical mechanisms at the level of ultra- and macrostructure [10]. Attempts to reconstruct the evolution of color-producing nanostructures using phylogenetic and/or structural evidence have hypothesized that multilayer structures in the scale lumen are evolutionarily primitive [11],[12], but these conclusions are not widely accepted [1],[10].

Fossils provide direct evidence of stages in the evolution of biological structures and can be used to test evolutionary hypotheses. The lepidopteran fossil record extends from the Early Jurassic to the Recent and includes representatives of numerous extant lepidopteran families [13],[14]. Fossil specimens of adult macrolepidopterans often exhibit light- and dark-toned areas on their wings [14] and can retain ultrastructural details of their scales [15]; preservation of pigmentary or structural colors has not been reported. Most fossil lepidopterans occur as inclusions in amber and within fine-grained sediments [14]; Baltic and Dominican amber (Eocene-Oligocene), the lacustrine sediments of Florissant (Eocene, Colorado), and the offshore marine sediments of the early Palaeocene Fur Formation (Denmark) are especially rich sources [14]. Fossil lepidopterans have also been reported (but not described) from the mid-Eocene Messel oil shale of Germany, which is celebrated for preserving a diverse paratropical ecosystem with remarkable fidelity [16]; the biota includes mammals, reptiles, amphibians, abundant fish and insects, and plants [17], the last represented by leaves, fruits, and seeds [18]. Messel fossils are typically well preserved: animals are often well-articulated and many show evidence of soft tissues (including stomach contents); insects (especially beetles) may exhibit metallic coloration [16]. Here we use scanning- and transmission electron microscopy (SEM and TEM), reflectance micro-spectrophotometry, and 2-D discrete Fourier analysis [1],[19] to demonstrate that metallic color in the fossil lepidopterans from Messel is structural in origin and to reconstruct their original color.

## Results and Discussion

**Figure 1:**
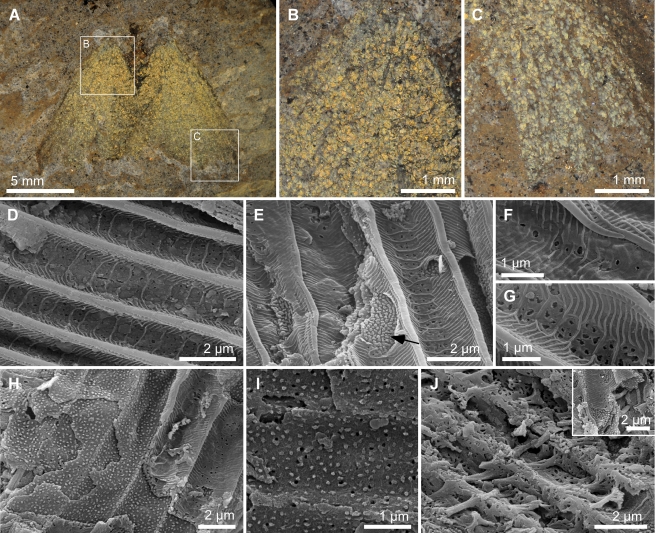
Structurally colored Messel (Eocene) lepidopterans. (A–C) Light micrographs of specimen MeI12269 with details of areas indicated (B, C). (D–J) Scanning electron micrographs of scales. (D) Surface showing longitudinal ridges and transverse crossribs and microribs. (E) Two overlapping scales showing windows, perforations, and internal laminae of the upper, fractured, scale. Arrow indicates densely packed bead- to rod-like spacers in the uppermost internal lamina. (F, G) Windows and perforations in proximal (F) and distal (G) parts of a scale. (H) Oblique fracture through scale showing successive internal laminae. (I) Surface of internal lamina showing perforations and bead-like spacers. (J) Horizontally fractured scale showing trabeculae (fractured and lying parallel to the scale surface) and reticulate basal lamina with, inset, intact vertically orientated trabeculae. Scale bars: (A), 5 mm; (B, C), 1 mm; (D, E, H, J) (including inset), 2 µm; (F, G, I), 1 µm.

The fossils (Table S1) occur as isolated individuals (Figure 1A, Figure S1) and in coprolites (Figure S1) but have not been described [20]. Wing venation patterns indicate that specimens are possibly extinct representatives of the Zygaenidae (burnet and forester moths), in particular Procridinae (forester moths; see Text S1). Two taxa are represented; specimens of the smaller taxon are more complete, and therefore the focus of this study. Electron dispersive X-ray analyses demonstrate that the fossil scales are organically preserved: they comprise predominantly carbon and there is no evidence for replacement of the preserved tissue by authigenic minerals.

Brilliantly colored scales cover the dorsal surface of the forewing except for a thin brown (non-metallic) zone along the outer margin (Figure 1A–C, Figure S1); they are restricted to basal and discal zones of the ventral surface (Figure S1). The dorsal surface of the hindwing is predominantly brown but exhibits metallic colors apically (Figure S1); the ventral surface is not visible in any specimen. Metallic scales also occur on the body of the insect. Specimens in glycerine exhibit predominantly yellow colors in basal and discal to postdiscal zones of the wing; the color grades to green and then blue in postdiscal to submarginal wing zones, and is brown along the outer wing margin (Figure 1A–C; Figure S1a–c). Scales on the abdomen typically exhibit yellow to orange colors in glycerine. The observed color varies when a fossil is placed in media of different refractive indices (Figure S2) in a fashion characteristic of many structurally colored materials [21].

### The Fossil Scales Preserve Diverse Anatomical Ultrastructures

**Figure 2:**
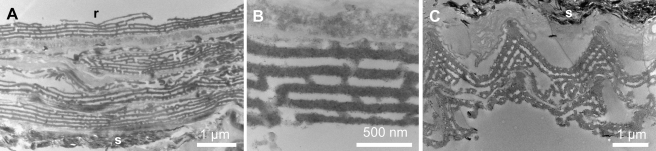
Transmission electron micrographs of the fossil multilayer reflector. (A) Vertical longitudinal section through a stack of four scales; scales are fractured locally. r, resin; s, sediment. (B) Detail of multilayer nanostructure in a longitudinal section through a single scale. (C) Transverse section through a scale showing broad ridges and the concave geometry of the interridge surface and of the underlying multilayer structure. Scale bars: (A, C), 1 µm; (B), 500 nm.

The gross morphology of the scales is difficult to determine as they overlap and are typically fractured. Ultrastructural evidence demonstrates that four types are present (Figures 1, 2, Figures S3, S4). Type A scales, the most common, are the primary contributor to the observed color. They are cover scales and occur over the dorsal and ventral surface of the forewing (Figures 1D–J, 2). The abwing surface of these scales, as in extant lepidopterans [22], exhibits prominent longitudinal ridges connected by orthogonal crossribs (typical spacing 1.8–2.5 µm and 510–600 nm, respectively) (Figure 1D). The ridges are up to 1 µm high (Figure 2C). They comprise overlapping lamellae (each 1.2–3.1 µm long and 110–150 nm wide) inclined at 10–12° to the scale surface and exhibit short lateral microribs (typical spacing 122–170 nm) (Figure 1D–G). The ridges and crossribs frame a series of windows that are typically perforated (Figure 1E–G); the lamina perforation factor (*p*) [23] increases from the proximal (*p* = 0.05) (Figure 1F) to distal (*p* = 0.32) parts of a scale (Figure 1G).

The scale lumen contains 3–5 perforated internal laminae that differ in their structure and thickness (Figures 1E,H, 2). The uppermost lamina (93–124 nm thick) exhibits densely packed, bead- to rod-like spacers (60 nm wide and 60–500 nm long) (arrow in Figure 1E). The next two to four laminae exhibit less densely packed, bead-like, spacers (typically 60 nm×60 nm) (Figure 1I) and decrease progressively in thickness (from 74–110 nm to 55–63 nm) towards the adwing scale surface (Figure 2A,B). In the proximal parts of a scale, the lowermost lamina in the stack is the basal lamina of the scale. In medial and distal parts of a scale, however, the stack is supported by additional pillar-like trabeculae (each 0.6–1 µm high) above a lamina with a distinctive reticulate texture (55–65 nm thick), which forms the base (Figure 1J). The ultrastructure of Type A scales (including the spacing of laminae, which is known to control color in living lepidopterans) varies according to their color and location on the wing (see Table S2). Similar variation occurs in extant lepidopterans [22],[24]. Even non-metallic brown scales in the fossils preserve ultrastructural details, including the laminar ultrastructure in the scale lumen (Figures S3, S5). Scale types B, C (“satin-type” [9]), and D are rare and do not contribute significantly to the observed color in the fossils (see Text S1).

Three-dimensional structures in fossils are vulnerable to compaction during burial of the host sediments. It is not assumed a priori that the preserved structure of the laminar array in the fossil lepidopteran scales is identical to that in vivo, especially as the trabeculae are typically fractured and now orientated parallel to, and superimposed upon, the basal lamina of the scale (Figure 2G). There is, however, no evidence that the laminar array has been similarly affected. Successive laminae are not superimposed and the vertical spacers between them are neither fractured nor flexed. Preferential fracturing of the trabeculae may have been promoted by their wider spacing and greater height. There is no evidence (e.g., dessication cracks) that the geometry of the laminar array was affected by shrinkage of the scales during diagenetic dehydration of the organic tissue. Nor is there evidence for diagenetic expansion of the scale structure: spacers are continuous between adjacent laminae. Collectively, these observations indicate that diagenetic processes had little or no impact upon the preserved structure of the laminar array. The preserved ultrastructure is therefore considered to be extremely similar, if not identical, to that originally present in vivo.

### The Laminar Array in the Scale Lumen Is a Fossilized Multilayer Reflector

**Figure 3:**
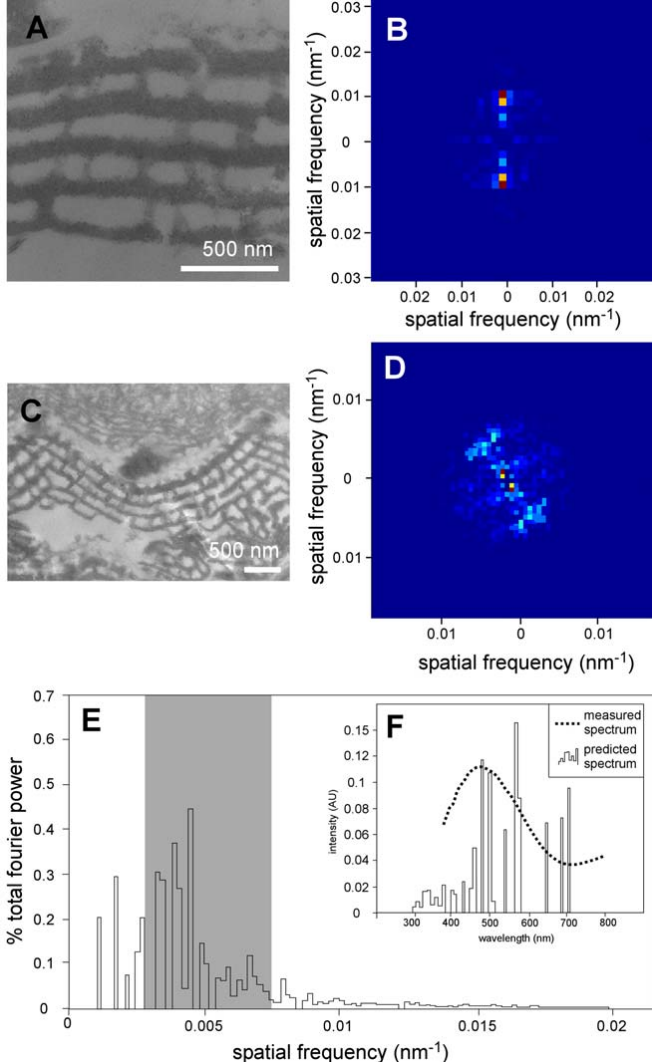
2-D Fourier analysis and reflectance microspectrophotometry of structurally colored scales from the basal part of the dorsal forewing. (A, C) Transmission electron micrographs of longitudinal (A) and transverse (C) sections. (B, D) 2-D Fourier power spectra of nanostructures in (A) and (C), respectively. Color scale (blue to red) indicates the relative magnitude of the squared Fourier components, which are dimensionless quantities. Direction from the origin indicates the direction of the 2-D component waves in the image, and the distance from the origin indicates the spatial frequency (cycles/nm) of each Fourier component. (E) Radial average of power spectrum in (B). Shaded area indicates the range of spatial frequencies that produces coherent scattering of visible wavelengths. (F) Measured and Fourier predicted reflectance spectra for nanostructure in (A). Scale bars: (A, C), 500 nm.

The ultrastructure of the laminar array was not modified during fossilization and is therefore a reliable basis for reconstructing the original colors of the fossil scales. 2-D Fourier analysis of longitudinal TEM images of the ultrastructure in the lumen of scales from the basal part of the dorsal forewing reveals two points of high values aligned above and below the origin (Figure 3A,B). The dominant periodicity is in the vertical direction; that is, the preserved structure is highly laminar. Fourier power spectra of transverse TEM images show a wider distribution of Fourier power peaks above and below the origin (Figure 3C,D). This results from the concave geometry of the laminar array in transverse section and consequent increase in the range of angles over which the observed color maintains the same peak hue [1]. Radial averages of the Fourier power spectra demonstrate that the preserved laminar nanostructure is a multilayer reflector: the peak spatial frequencies in refractive index lie within the range capable of producing visible colors by scattering of light (Figure 3E).

The visual properties of extant lepidopteran scales can be influenced by scale tilt (the angle between the scale and the wing membrane) [10], scale curvature [2], the number and thickness of laminae [25], the degree of overlap of ridge lamellae [21], the spacing of the ridges [21], microribs [21], and crossribs [26], and the lamina perforation factor [23]. Scale tilt and curvature are not preserved in the fossils. The number (up to five) of laminae and their different thicknesses indicate that the fossil multilayer reflector is non-ideal (i.e. reflects much less than 100% of incoming light) [27]. The ridge lamellae in the fossils do not overlap sufficiently [28] to have a significant impact on the observed color. Closely spaced microribs or crossribs in satin-type scales can also generate diffraction [26] and play a secondary role in the generation of blue and violet colors in lepidopteran scales [19],[23]. The spacing of the microribs (140 nm) in the fossil satin-type scales, however, is significantly less than the wavelength of visible light (approx. 350–700 nm); conventional diffraction theory indicates that zero-order diffraction (i.e. specular (directional) reflectance) will be produced. Further, the satin-type scales are restricted to the inner margin of the forewing in the fossils, precluding their having a significant impact on the observed color. Collectively, these observations indicate that the primary color-producing nanostructure in the fossils is the multilayer reflector in the scale lumen.

The optical properties of the fossil scales are, however, influenced by the perforation factor and concave geometry of the laminae, and by the spacing of the ridges. In extant lepidopterans, iridescence, spectral bandwidth, and total reflectance are reduced at higher perforation factors (between 0.2 and 0.4) relative to scales with lower perforation factors [23]; this generates a purer (albeit less intense) color that is visible over a wider range of angles. In the fossils the exposed (medial and distal) parts of the overlapping scales typically have perforation factors of 0.32. Concave distortion of laminar arrays also reduces iridescence [1]; the arcuate geometry of the fossil multilayer reflector in transverse section would have enhanced the iridescence-reducing effect of the perforated laminae. Multilayer reflectors typically generate directional (specular) color that flashes at specific observation angles [29]; this effect can be modified by diffraction. Ridge periodicities of between 0.85 µm and 4 µm generate diffraction [21],[30],[31]; a strong diffractive effect has been reported for periodicities of ca. 1.3 µm [31] and 1.7 µm [30]. The ridges in the fossils are spaced 1.8–2.5 µm apart and therefore probably constitute diffraction elements that render the color generated by the multilayer reflector visible over a wide range of observation angles, but do not contribute to the observed hue [31].

### The Original Colors of the Fossils Are Not Preserved But Can Be Reconstructed

**Figure 4:**
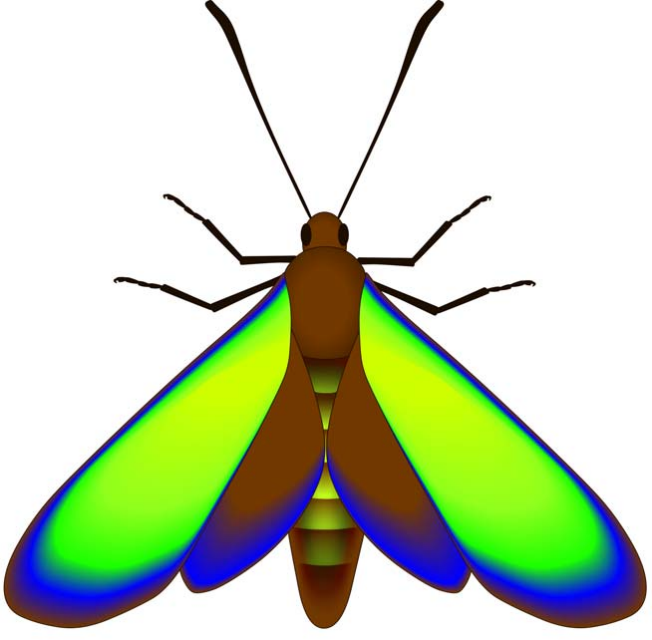
Reconstruction of the original colors of the dorsal surface of the fossil lepidopterans. The dominant hue of basal and discal to postdiscal zones of the wing is blue today (in air) but was originally yellow-green. Postdiscal to submarginal wing zones were originally green-cyan and blue, and the outer wing margin, brown.

Scales from the dorsal surface of the basal part of the forewing exhibit a measured reflectance peak of 473 nm (Figure 3F) that corresponds to their blue color in air. The predicted peak of reflectance (with λ_max_ = 565 nm) calculated from the radial averages (using refractive index values of 1.56 and 1.0 for the high- and low-index layers, respectively), however, indicates that the dorsal surface of the basal part of the forewing was originally yellow-green. The color in air today and the measured reflectance peak are artefacts, probably a result of alteration of the biomolecular composition of the scale cuticle, and thus its refractive index, during fossilization; most fossil arthropod cuticles are chemically altered during diagenesis [32]. Furthermore, recent experiments using extant butterfly scales demonstrated that alteration of the original organic material results in a shift in the reflectance peak without altering the scale ultrastructure significantly [33]. Calculation of reflectance peaks for scales from other parts of the wings (see Figure S5) allows the original colors of the fossil lepidopterans to be reconstructed (Figure 4; Table S3). Scales in postdiscal to submarginal wing zones have predicted reflectance peaks λ_max_≈515 nm and λ_max_≈440 nm, respectively; scales along the wing margins have a predicted reflectance peak λ_max_≈750 nm, and scales from the abdomen have a predicted reflectance peak λ_max_≈550 nm (Figure S5). The fossil lepidopterans therefore originally exhibited yellow-green hues in basal and discal to postdiscal zones of the wing; the color graded to green-cyan and then blue in postdiscal to submarginal wing zones, and was brown along the outer wing margin. Scales on the abdomen were yellow to yellow-green.

### Functional Ecology of the Structural Color

Structural colors in extant butterflies function primarily in species and mate recognition [29]; the function of structural colors in extant moths, however, has not been investigated. The fossil moths described here are colored most highly on the dorsal surfaces of the forewings (the surfaces which are exposed in most extant moths, including zygaenids, when they are at rest [34]), suggesting that the Eocene moths, like extant zygaenids, were diurnal. The visual ecology of the structural color in the fossil moths can therefore be compared with those in extant diurnal lepidopterans. The fossil moths were characterized by a yellow-green dorsal coloration that was visible over a wide range of angles but not highly reflective. The visual signal lacked certain properties, e.g. strong iridescence, brightness, and color contrast within the wing, that are important in conspecific communication [30]. Instead, the optical characteristics of the fossil scales, notably their original yellow-green hue and suppression of iridescence, indicate a primary defensive function. In extant lepidopterans, reduced iridescence enhances presentation of visual signals for protective purposes [35]. Structural green coloration functions cryptically in extant butterflies [24],[29] and beetles [36],[37]. In particular, a combination of a structural green hue with reduced iridescence provides particularly efficient color matching with a diffuse leafy background [36],[37].

A cryptic function for the structural color in the fossil lepidopterans is consistent with the ecology of extant zygaenid moths: many Procridinae species with green scales are cryptic except when feeding on flowers [38], when they can be highly conspicuous (Gerhard Tarmann (Tirolier Landesmuseen, Austria), personal communication) [39]. The latter feature is inconsistent with cryptism: high chromatic contrast with the background environment is characteristic of an aposematic (warning) signal [40]. However, an aposematic function for the structural color while feeding does not necessarily conflict with a cryptic function in a foliaceous environment: dual-purpose visual signals are known in extant lepidopterans [41]–[43]. The visual signal generated by the structurally colored scales in the fossil lepidopterans probably served two functions: cryptic when specimens were at rest, and aposematic during nectaring. It is possible that this dual function is evolutionarily conserved in Procridinae and that aposematism and diurnality are ancestral traits of zygaenids. Further, defensive behavior in the fossil moths is consistent with the use of chemical defense: extant zygaenids, including taxa that are largely cryptic [28], can synthesize cyanide for defense by enzymatic breakdown of cyanoglucosides [24],[44],[45].

### Wider Implications

The discovery of structural color in Messel lepidopterans constrains the timing of the origin of several important evolutionary novelties. Different scale types in extant lepidopterans arise via subtle modifications of a common membrane-folding developmental process dominated by self-assembly [9],[22],[46]. The presence of different scale types in the fossils confirms that such plastic developmental processes had evolved in moths by the mid-Eocene. The complexity of the iridescence-reducing nanostructure in the fossil moths indicates that sophisticated optical mechanisms for interspecific signaling were in use at this time. Predator-prey interactions are recognized as a major stimulus in insect evolution [47]; the use of cryptic and aposematic signals by the fossil moths described here supports the evidence of other fossils from Messel [48] that sophisticated mechanisms for avoiding detection by visually hunting predators had evolved in insects by the mid-Eocene. The striking resemblance of the fossil moths to some extant zygaenids and the cryptic/aposematic function of their structural color suggest that dual-purpose visual signals, and especially aposematism, may be evolutionarily conserved in this group of moths, originating early in the history of the group and persisting to the present day. Preservation of ultrastructural detail in all scales in the fossils, even non-metallic brown examples, offers the possibility of reconstructing the original colors and patterning of even lepidopteran fossils that lack obvious structural color.

## Materials and Methods

### Fossil Material

Specimens are held by the Senckenberg Forschungsinstitut und Naturmuseum, Forschungsstation Grube Messel, Germany.

### SEM and TEM Analysis

Small (2–3 mm^2^) tissue samples were removed using sterile tools and, for TEM, placed in the following ethanol∶glycerine mixtures, each for 24 h under rotation: 10%, 25%, 50%, 75%, 100% ethanol. For SEM, samples were dehydrated using HMDS or under vacuum, mounted on aluminum stubs, carbon- or gold-coated, and examined using a FEI XL-30 ESEM-FEG microscope equipped with an EDAX energy disperse X-ray spectrometer. Observations were made at an accelerating voltage of 15 kV, with acquisition times of 60 s for EDS spectra of carbon-coated samples. For TEM, samples were washed in propylene oxide twice, each for 1 h, and impregnated with Spurr's resin under vacuum in the following resin∶ethanol mixtures, each for 24 h: 10%, 20%, 30%, 40%, 50%, 60%, 70%, 80%, 90%, 100% resin. To ensure optimal orientation for sectioning, a 10 mm^3^ block of resin containing the sample was extracted, re-orientated, and re-embedded in 100% resin. Ultrathin (80–90 nm thick) microtome sections were placed on formvar-coated Cu grids, stained using uranyl acetate and lead citrate, and examined using a Zeiss EM900 TEM at 80 kV with an objective aperture of 90 µm diameter.

### Reflectance Microspectrophotometry

Reflectance spectra were recorded from samples in 100% glycerine, 100% ethanol, and in air (the latter from only the basal part of the dorsal forewing to minimize damage due to drying) using an epi-illumination Nikon Optiphot 66 microscope, an Ocean Optics HR2000+ spectrophotometer, and a tungsten-halogen light source; spectra were collected from a 70 µm spot. All recorded spectra were normalized against the spectrum of the light source recorded from a white standard.

### 2-D Fourier Analysis

Nanoscale spatial periodicity in the refractive index of a material results in constructive interference of scattered light; structural color is generated where such scattering occurs in the visible part of the spectrum. Herein we use an established analytical method [13] of analyzing the periodicity and optical properties of structurally colored biological tissues using the discrete Fourier 2-D transform. Digital TEM micrographs of scales from the fossil lepidopterans were analyzed using MATLAB (version 7.11.0) and a 2-D Fourier tool freely available as a series of MATLAB commands (http://www.yale.edu/eeb/prum/fourier.htm). Variation in the refractive index of nanostructures in the fossil scales was analyzed using the procedure described in ref. [1].

### Reconstruction of Original Colors

The reconstruction of the original colors of the fossil lepidopterans is based upon the preserved ultrastructure of the multilayer reflector in the scale lumen and the assumption that the original refractive index of the fossil scale cuticle was similar to that in modern lepidopterans (i.e. ∼1.56). Only the original colors of the dorsal surface of the specimens were reconstructed: (1) only this surface is exposed at rest and (2) the ventral surface of the hindwing is not visible in any specimen. TEM images of the multilayer reflector preserved in Type A scales of different colors were analyzed using 2-D Fourier analysis. Predicted wavelength values scales from different locations on the wing are based on 2–4 replicate analyses. Wavelength data for predicted reflectance peaks were converted to RGB values. Calculation of precise RGB values for a specific wavelength, however, is difficult [49]. RGB values for predicted wavelength data were therefore calculated using three different methods: (1) using the “Wavelength to RGB” application available from http://miguelmoreno.net/sandbox/wavelengthtoRGB/ (downloaded December 28, 2010), (2) using the “Spectra” application available from www.efg2.com/lab (downloaded December 28, 2010), and (3) using the “Wavelength to RGB” converter available online at www.uvm.edu/~kspartal/Physlets/Lecturedemo/LambdaToRGB.html (accessed December 28, 2010). The three methods yield similar RGB values; the colors depicted in the reconstruction are based on the averages of the values obtained.

## Supporting Information

Figure S1 Structurally colored lepidopteran fossils and forewing venation patterns. (a–d) Light micrographs of specimen MeI 11792 (a), MeI 14861 (b), MeI 641 (c), and MeI 11808 (d, e) (a coprolite). Note that the forewings are incomplete in MeI 11792. (e) shows detail of area indicated in (d). (f) Reconstruction of the forewing venation based on specimens MeI 641 and MeI13556, with nomenclature of the wing veins. A, anal; CuA, anterior cubitus; CuP, posterior cubitus; M, media; R, radius; Rs, radial sector; Sc, subcosta. Scale bars: (a–d), 10 mm; (e), 2 mm; (f), 1 mm.
(TIF)

Figure S2 Variation in observed color and in reflectance spectra of scales in media of different refractive index. (a, b) show the same area from the basal forewing of specimen MEI 14861. Scales appear yellow-orange when in glycerine (a) and blue in air (b). (c) Measured reflectance spectra of scales from the area shown in (a, b). Peak wavelength is 603 nm in glycerine and 473 nm in air. Scale bars in (a, b), 250 µm.
(TIF)

Figure S3 Scanning electron micrographs of fossil lepidopteran forewing scales and wing membrane. (a) Basal region of Type A scale from discal zone of the wing, showing closely packed microribs and absence of windows. (b) Surface of brown non-metallic Type A scale from the outer margin of the wing, showing ridges, microribs, and perforations. (c) Transverse fractured section through Type B (cover) scale (B) and Type D (ground) scale (D) from discal part of the forewing. Note wing membrane (W) underlying ground scale. (d) Detail of area indicated in (c), showing weakly laminar nanostructure in the lumen of the Type D scale, and well-defined laminar structure in the lumen of the Type B scale. Note the granular layer underlying the laminar structure in the Type B scale. (e) Surface of Type D scale. (f) Surface of Type C (cover) “satin” scale from the inner margin of the discal zone of the wing, showing closely spaced microribs. (g) Transverse fractured section through Type C scales, showing granular texture in scale lumen. (h) Type A scale from coprolite, showing surficial ridges and microribs, laminae in scale lumen, and basal reticulate lamina. (i) Wrinkled texture of wing membrane. Scale bars: (a), 5 µm; inset in (a), 1 µm; (b–h), 2 µm; (i), 10 µm.
(TIF)

Figure S4 Schematic reconstructions of the various scale types preserved in the fossil lepidopterans. (a) Type A scale showing longitudinal ridges (R) with transverse crossribs (C) and microribs (M) on the scale surface; the scale lumen comprises a stack of perforated laminae underlain by trabeculae (T). Note perforations (P) in, and bead-like and rod-like spacers on, each lamina. (b) Type B scale. The stack of laminae is underlain by granular material; trabeculae are absent. (c) Type C scale showing closely spaced crossribs; the lumen comprises granular material. (d) Type D scale showing poorly defined microribs and laminae in the scale lumen.
(TIF)

Figure S5 Ultrastructure and predicted wavelength of Type A scales of different color and from different locations on the wing. (a, c, e) Transmission electron micrographs of scales from submarginal (a), postdiscal (c), and outer marginal (e) wing zones, and from the abdomen (g). Scales appear blue (a), green (b), brown (e), and yellow-orange (g) in glycerine. (b, d, f, h) Fourier predicted reflectance spectrum for the nanostructures in (a), (c), (e), (g), respectively. Predicted reflectance peak is ∼440 nm in (b), ∼515 nm in (d), ∼750 nm in (f), and ∼550 nm in (h). Scale bars: (a, c, e, g), 500 nm.
(TIF)

Table S1 List of specimens studied.
(PDF)

Table S2 Ultrastructural details of scales exhibiting different colors and (in the case of intact individuals) from different parts of the forewing.
(PDF)

Table S3 Conversion of wavelength data for predicted reflectance peaks to RGB values.
(PDF)

Text S1 Systematic paleontology; color and reflectance spectra of scales in media of different refractive index; preserved scale types and other ultrastructural features; and supplementary references.
(DOC)
