# Supplementary figures and images for: Fossilized Biophotonic Nanostructures Reveal the Original Colors of 47-Million-Year-Old Moths
Source: PLoS Biol. 2011 Nov 15;9(11):e1001200. doi: 10.1371/journal.pbio.1001200 (PMC3217029; doi:10.1371/journal.pbio.1001200)

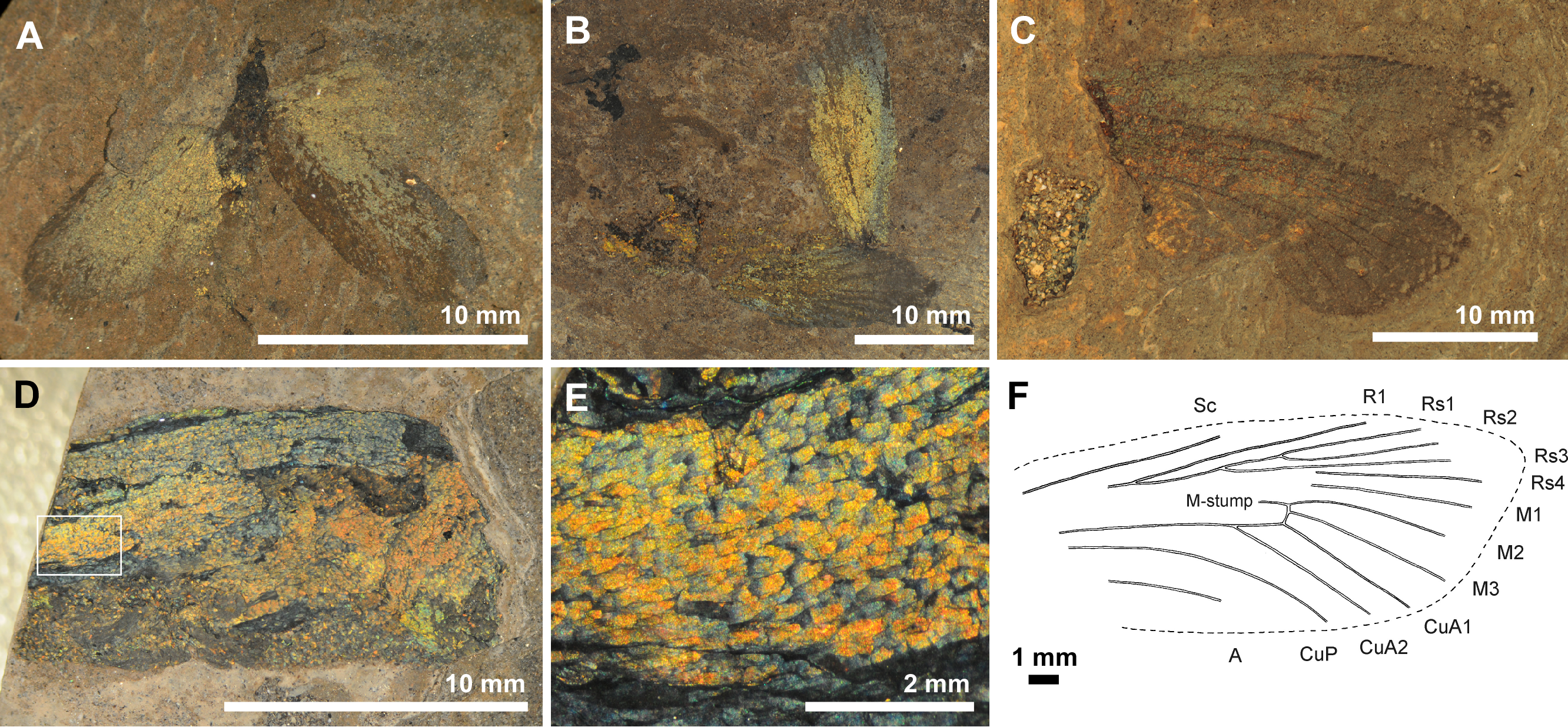

Supplement: Figure S1 — Structurally colored lepidopteran fossils and forewing venation patterns. (a–d) Light micrographs of specimen MeI 11792 (a), MeI 14861 (b), MeI 641 (c), and MeI 11808 (d, e) (a coprolite). Note that the forewings are incomplete in MeI 11792. (e) shows detail of area indicated in (d). (f) Reconstruction of the forewing venation based on specimens MeI 641 and MeI13556, with nomenclature of the wing veins. A, anal; CuA, anterior cubitus; CuP, posterior cubitus; M, media; R, radius; Rs, radial sector; Sc, subcosta. Scale bars: (a–d), 10 mm; (e), 2 mm; (f), 1 mm. (TIF) [file pbio.1001200.s001.tif]

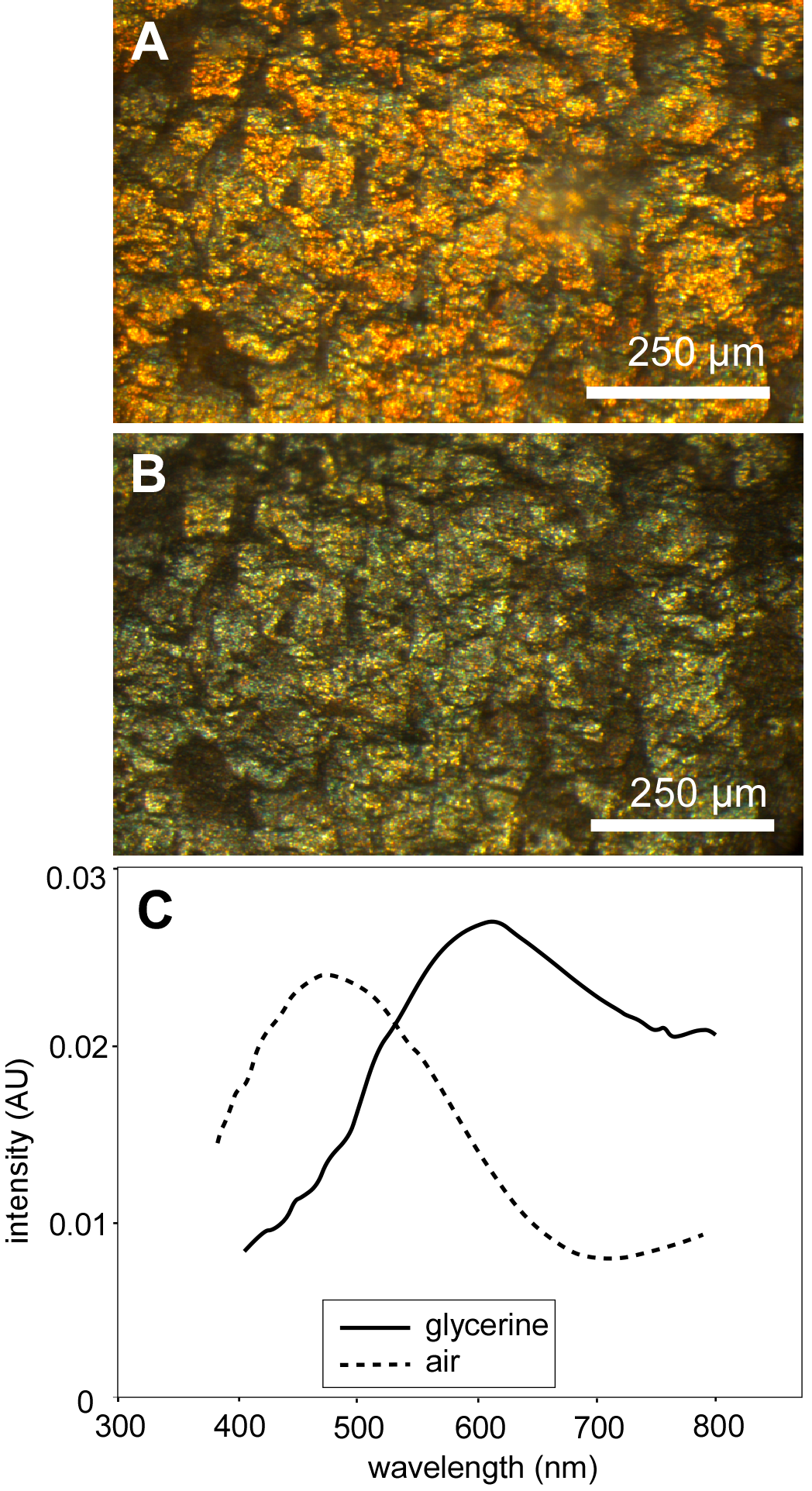

Supplement: Figure S2 — Variation in observed color and in reflectance spectra of scales in media of different refractive index. (a, b) show the same area from the basal forewing of specimen MEI 14861. Scales appear yellow-orange when in glycerine (a) and blue in air (b). (c) Measured reflectance spectra of scales from the area shown in (a, b). Peak wavelength is 603 nm in glycerine and 473 nm in air. Scale bars in (a, b), 250 µm. (TIF) [file pbio.1001200.s002.tif]

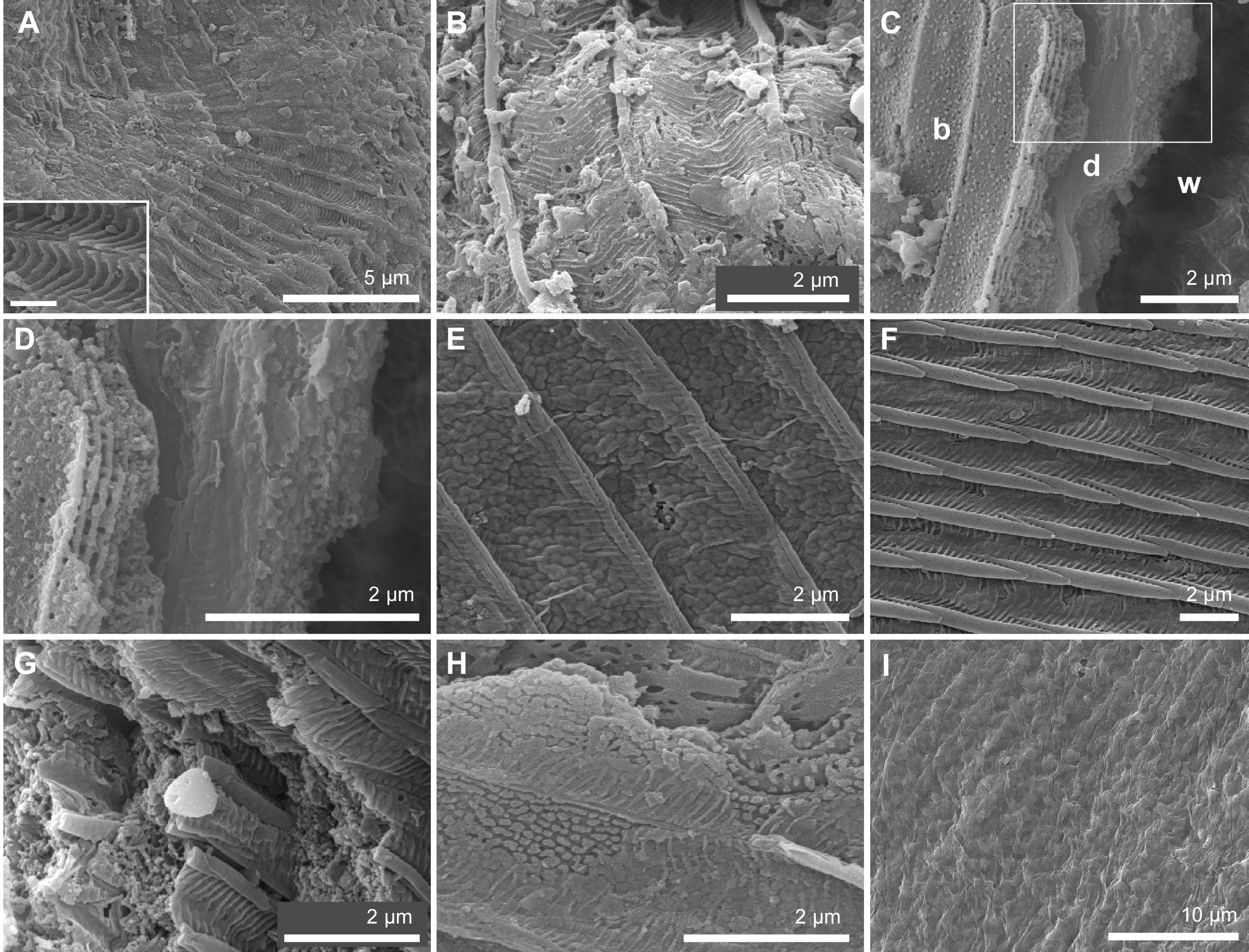

Supplement: Figure S3 — Scanning electron micrographs of fossil lepidopteran forewing scales and wing membrane. (a) Basal region of Type A scale from discal zone of the wing, showing closely packed microribs and absence of windows. (b) Surface of brown non-metallic Type A scale from the outer margin of the wing, showing ridges, microribs, and perforations. (c) Transverse fractured section through Type B (cover) scale (B) and Type D (ground) scale (D) from discal part of the forewing. Note wing membrane (W) underlying ground scale. (d) Detail of area indicated in (c), showing weakly laminar nanostructure in the lumen of the Type D scale, and well-defined laminar structure in the lumen of the Type B scale. Note the granular layer underlying the laminar structure in the Type B scale. (e) Surface of Type D scale. (f) Surface of Type C (cover) “satin” scale from the inner margin of the discal zone of the wing, showing closely spaced microribs. (g) Transverse fractured section through Type C scales, showing granular texture in scale lumen. (h) Type A scale from coprolite, showing surficial ridges and microribs, laminae in scale lumen, and basal reticulate lamina. (i) Wrinkled texture of wing membrane. Scale bars: (a), 5 µm; inset in (a), 1 µm; (b–h), 2 µm; (i), 10 µm. (TIF) [file pbio.1001200.s003.tif]

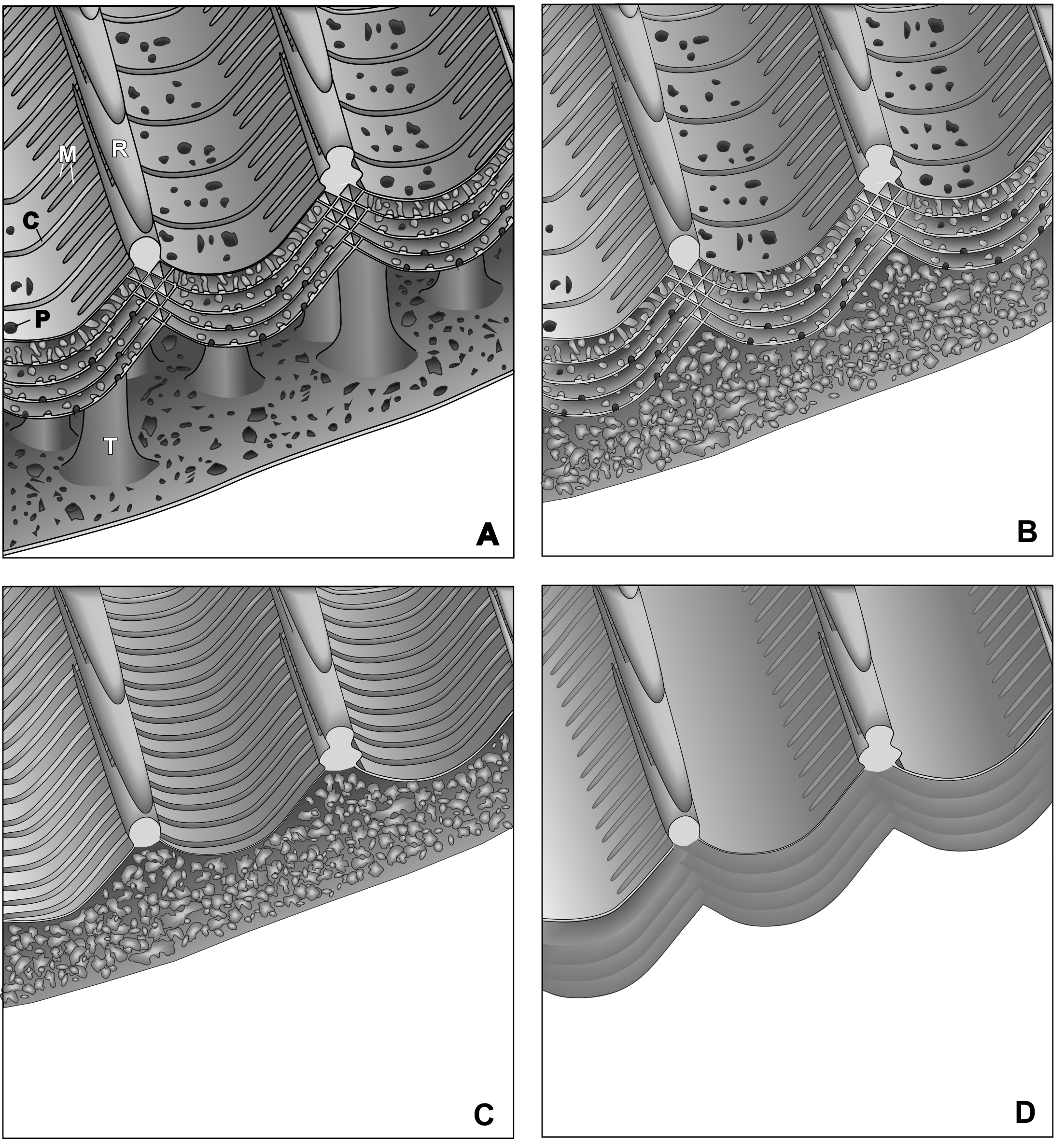

Supplement: Figure S4 — Schematic reconstructions of the various scale types preserved in the fossil lepidopterans. (a) Type A scale showing longitudinal ridges (R) with transverse crossribs (C) and microribs (M) on the scale surface; the scale lumen comprises a stack of perforated laminae underlain by trabeculae (T). Note perforations (P) in, and bead-like and rod-like spacers on, each lamina. (b) Type B scale. The stack of laminae is underlain by granular material; trabeculae are absent. (c) Type C scale showing closely spaced crossribs; the lumen comprises granular material. (d) Type D scale showing poorly defined microribs and laminae in the scale lumen. (TIF) [file pbio.1001200.s004.tif]

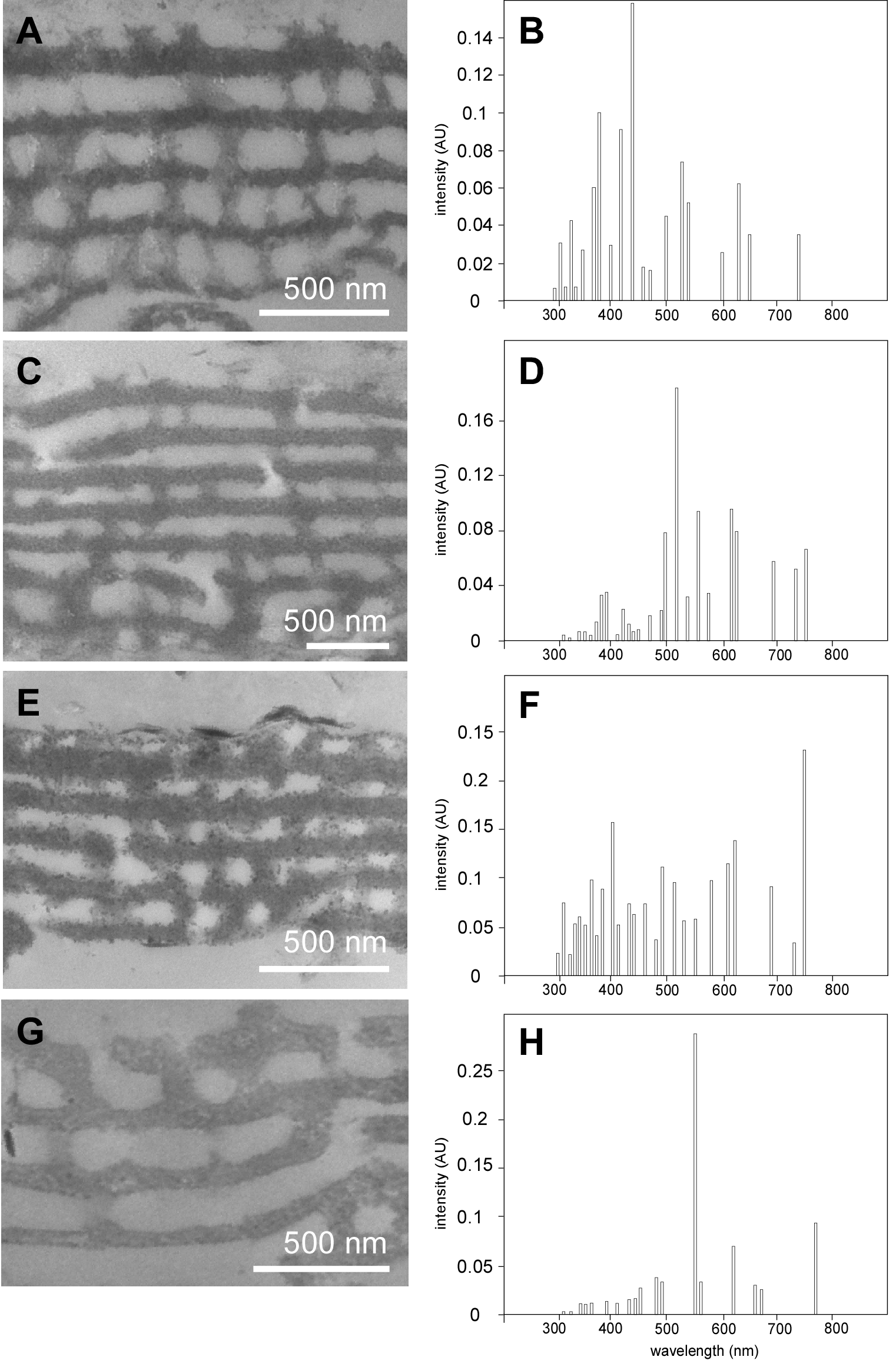

Supplement: Figure S5 — Ultrastructure and predicted wavelength of Type A scales of different color and from different locations on the wing. (a, c, e) Transmission electron micrographs of scales from submarginal (a), postdiscal (c), and outer marginal (e) wing zones, and from the abdomen (g). Scales appear blue (a), green (b), brown (e), and yellow-orange (g) in glycerine. (b, d, f, h) Fourier predicted reflectance spectrum for the nanostructures in (a), (c), (e), (g), respectively. Predicted reflectance peak is ∼440 nm in (b), ∼515 nm in (d), ∼750 nm in (f), and ∼550 nm in (h). Scale bars: (a, c, e, g), 500 nm. (TIF) [file pbio.1001200.s005.tif]
