# Supplementary material for: Fossilized Biophotonic Nanostructures Reveal the Original Colors of 47-Million-Year-Old Moths
Source: PLoS Biol. 2011 Nov 15;9(11):e1001200. doi: 10.1371/journal.pbio.1001200 (PMC3217029; doi:10.1371/journal.pbio.1001200)
Supplement: Table S1 — List of specimens studied. (PDF) [file pbio.1001200.s006.pdf]

prefix	*catalogue number*
Mel	296
Mel	641
Mel	650
Mel	3046
Mel	3552
Mel	5944
Mel	10179
Mel	11792
Mel	11808
Mel	11851
Mel	12046
Mel	12113
Mel	12215
Mel	12228
Mel	12269
Mel	12311
Mel	12328
Mel	12460
Mel	12474
Mel	13556
Mel	14034
Mel	14093
Mel	14145
Mel	14199
Mel	14201
Mel	14253
Mel	14293
Mel	14488
Mel	14496
Mel	14553

Table S1: List of specimens studied. All specimens are held by the Senckenberg Forschungsinstitut und Naturmuseum, Forschungsstation Grube Messel.
