# Supplementary material for: Fossilized Biophotonic Nanostructures Reveal the Original Colors of 47-Million-Year-Old Moths
Source: PLoS Biol. 2011 Nov 15;9(11):e1001200. doi: 10.1371/journal.pbio.1001200 (PMC3217029; doi:10.1371/journal.pbio.1001200)
Supplement: Table S2 — Ultrastructural details of scales exhibiting different colors and (in the case of intact individuals) from different parts of the forewing. (PDF) [file pbio.1001200.s007.pdf]

Table S2: Ultrastructural details of scales exhibiting different colours and (in the case of intact individuals) from different parts of the forewing

Specimen	*Type of specimen*	*Location of sample*	*Scale colour in glycerine*	*Scale thickness (um)*	*Ridge spacing (um)*	*Cross rib spacing (nm)*	*Microrib spacing (nm)*	*Ridge lamella length (um)*	*Ridge lamella overlap*	*No. of laminae in lumen*
14861	intact individual	basal/discal	yellow	1.48	1.9-2.5	600	138-150	1.35	2	4-8
14861	intact individual	basal/discal	yellow-orange	1.27	1.8-2.6	550-610	175	2.9-3.1	2-3	6
14861	intact individual	postdiscal	green	-	2.6	-	122	-	-	≤ 5
14861	intact individual	submarginal	blue	0.16 - 0.87	2.07-2.82	580	176	2.1	2-3	4
14861	intact individual	wing margin	brown	0.25	-	-	-	-	-	-
11808	coprolite	-	yellow	1.2	2.6	540	133-188	1.18	3-4	≤ 7
11808	coprolite	-	blue	1.25	1.9-2.2	530	172	2.9-3.2	3-4	≤ 7

Perforation factor: abbreviations refer to position on the scale as follows: d, distal; m, medial; p, proximal.

perforation factor	Details of laminae in scale lumen								Lamina thickness (nm)							
	1: microribs+crossribs	***2: large rod-like spacers***	***3: bead-like spacers***	***4: bead-like spacers***	***5: bead-like spacers***	***6: bead-like spacers***	***7: trabecular layer***	***8: granular base layer***	***1***	***2***	***3***	***4***	***5***	***6***	***7***	***8***
0.05(p)-0.32(d)	y	y	y	y	y	y	y	y	40	124	110	87	72	66	59	-
0.05(p)-0.3(d)	y	y	y	y	y	n	y	y	-	98	89	81	77	63	55	47
-	y	y	y	y	-	-	-	-	-	-	-	-	-	-	-	-
0.15(p)	y	y	y	n	n	n	y	y	31	111	79	-	-	-	63	31
-	y	y	y	n	n	n	n	y	15	111	45	-	-	-	-	42
0.2(m)-0.32(d)	y	y	y	y	y	y	y	y	111	93	74	67	60	53	-	35
0.25 (d)	y	y	y	y	y	y	y	-	31	111	87	63	55	47	-	-
