# Supplementary material for: Fossilized Biophotonic Nanostructures Reveal the Original Colors of 47-Million-Year-Old Moths
Source: PLoS Biol. 2011 Nov 15;9(11):e1001200. doi: 10.1371/journal.pbio.1001200 (PMC3217029; doi:10.1371/journal.pbio.1001200)
Supplement: Table S3 — Conversion of wavelength data for predicted reflectance peaks to RGB values. (PDF) [file pbio.1001200.s008.pdf]

Table S3: Conversion of wavelength data for predicted reflectance peaks to RGB values.

		Method 1			Method 2			Method 3			Average		
location of scales	predicted wavelength	R	G	B	R	G	B	R	G	B	R	G	B
submarginal	440	3	0	255	0	0	255	0	0	255	1	0	255
postdiscal - submarginal	515	15	255	0	31	255	0	31	255	0	25.7	255	0
basal & discal	565	197	255	0	210	255	0	210	255	0	205.7	255	0
wing margins	750	143	0	0	161	0	0	161	0	0	155	0	0
abdomen	550	156	255	0	163	255	0	163	255	0	160.7	255	0

Method 1: "Wavelength to RGB" application available from http://miguelmoreno.net/sandbox/wavelengthtoRGB/ (downloaded 28/12/2010)
Method 2: "Spectra" application available from www.efg2.com/lab (downloaded 28/12/2010)
Method 3: "Wavelength to RGB converter" online at www.uvm.edu/~kspartal/Physlets/Lecturedemo/LambdaToRGB.html (accessed 28/12/2010)
