# Supplementary material for: Fossilized Biophotonic Nanostructures Reveal the Original Colors of 47-Million-Year-Old Moths
Source: PLoS Biol. 2011 Nov 15;9(11):e1001200. doi: 10.1371/journal.pbio.1001200 (PMC3217029; doi:10.1371/journal.pbio.1001200)
Supplement: Text S1 — Systematic paleontology; color and reflectance spectra of scales in media of different refractive index; preserved scale types and other ultrastructural features; and supplementary references. (DOC) [file pbio.1001200.s009.doc]

**Fossilized Biophotonic Nanostructures Reveal the Original Colors of 47 Million-year-old Moths – Supporting Text**

Maria E. McNamara*^1,2^, Derek E.G. Briggs^1,3^, Patrick J. Orr^2^, Sonja Wedmann^4^, Heeso Noh^5^ & Hui Cao^5^

^1^Dept. of Geology & Geophysics, Yale University, New Haven, CT 06511, USA. ^2^UCD School of Geological Sciences, University College Dublin, Belfield, Dublin 4, Ireland. ^3^Yale Peabody Museum of Natural History, Yale University, New Haven, CT 06520, USA. ^4^Senckenberg Forschungsinstitut und Naturmuseum, Forschungsstation Grube Messel, D-64409 Messel, Germany. ^5^Dept. of Applied Physics, Yale University, New Haven, CT 06511, USA.

*Email: [maria.mcnamara@yale.edu](mailto:maria.mcnamara@yale.edu)

**Systematic palaeontology**

Lepidoptera Linnaeus, 1758

Ditrysia Borner, 1925

?Apoditrysia Borner, 1925

?Zygaenoidea Fracker, 1915

?Zygaenidae Latreille, 1809

**Horizon and locality.** The lepidopteran fossils were collected at the Messel Pit near Darmstadt (Hesse, Germany). All specimens are from the Messel Formation (lower Middle Eocene, lowermost Geiseltalian, Mammal Palaeogene level 11); specimens were recovered from various grid squares of the pit and at various stratigraphic levels. The fossils are hosted within organic-rich, laminated mudstones that were deposited in a deep, stratified, maar lake^50,51^. The fossiliferous sediments are ~47 million years old^52^.

**Material.** Specimens (Table S1) occur as isolated individuals (Figure 1a, Figure S1a-c) and within coprolites (Figure S1d, e). Individual specimens are usually incomplete and/or disarticulated. Coprolites comprise masses of densely packed scales that are randomly orientated or aligned locally (Figure S1d, e); other lepidopteran anatomical details are not evident. The producer of the coprolites is unknown; the structurally coloured scales are not associated with other, diagnostic, faecal material. Except where stated otherwise, all further discussion of specimens and their scales relates to individuals (not coprolites).

**Diagnosis.** Specimens belong to one of two size categories based on the length of the forewing (Group 1: 11-16 mm (Figure 1a, Figure S1a, b); Group 2: 25-27 mm (Figure S1c)) and are therefore unlikely to be conspecifics. The similarity of the preserved colour and ultrastructure of the scales of specimens from each group, however, indicates a close systematic relationship between the two groups.

The absence of microtrichia between the scales of the forewings of specimens from both groups indicates a position among Ditrysia^53^. It was not possible to reconstruct wing venation patterns for specimens from Group 1, or for the hindwing of specimens from Group 2, as the veins are typically obscured (by the scales or where the forewing is superimposed upon the hindwing), incompletely preserved, or the wing is missing). The forewing venation of specimens from Group 2 was reconstructed based on two specimens (MeI 641 and MeI 13556) (Figure S1f). The unbranched subcostal vein (Sc) indicates a position among Heteroneura. The discal cell is present, the distalmost part of a median vein (M-stump) is present, veins Rs1+Rs2 are fused basally, and vein Rs4 is postapical in position. A similar combination of characters is found in extant Zygaenidae: Procridinae^54^. It is challenging to infer systematic affinities of lepidopterans on the basis of the forewing venation alone due to the homoplasous nature of lepidopteran venational features. The gross visual appearance of the fossils, and the preserved ultrastructure of the scales, however, are consistent with a placement of the fossils within the Zygaenidae. Extant zygaenids are highly conspicuous moths in which the wings typically exhibit a striking metallic sheen; in particular, most members of the Procridinae exhibit a (near-) unicolorous metallic sheen on the dorsal surface of the forewing^34^. This feature also characterises the fossil specimens. In extant zygaenids, metallic scales can occur on other body parts (in addition to the wings), e.g. the head, thorax, abdomen, legs and antennae. Fossil specimens in both groups exhibit metallic scales on the abdomen, and specimens in group 2 also exhibit metallic scales on the thorax, legs, and the antennae. The ultrastructure of extant zygaenid scales includes perforated, concave laminar arrays^54,55^, laminar arrays underlain by trabeculae^21^, and ‘satin-type’^22^ scales (see below)^54,55^, each of which occur in the fossils.

**Colour and reflectance spectra of scales in media of different refractive index.**

The apparent colour of structurally coloured tissues comprising a matrix of a biomaterial (e.g. chitin) and air alters when the tissue is placed in media of different refractive index due to the replacement of air by the substitute medium in the matrix; this is a simple test for the presence of structural colour^15^. This approach was applied to the Messel lepidopterans as follows. The standard storage medium for fossils from Messel is glycerine (100%, or 70-99% in water). To investigate the effect of the refractive index of the surrounding medium upon the colour of the lepidopteran scales, a single specimen was placed successively in the following media, each for approximately one minute: 100% glycerine (R.I. = 1.45) and air (R.I. = 1.0) (the specimen was subsequently returned to the storage medium). Scales from the basal forewing were photographed, and their reflectance spectra measured, in each medium (Figure S2). λ_max_ values are 603 nm in glycerine and 473 nm in air, corresponding to observed yellow-orange and blue colours. Subtle variations in colour within an individual scale reflects its uneven topography due to subtle deformation of the scale surface (e.g. adjacent to sedimentary particles) during compaction; it is not true opalescence.

**Preserved scale types and other ultrastructural features**

Scales are preserved within individual fossils (on the wings and, to a lesser extent, other body parts (see above) and coprolites. The density and distribution of scales on the head, thorax, limbs and antennae of specimens in Group 1 cannot be determined accurately as these body parts are frequently obscured in part by the wings or sediment, or are incomplete. The following scale types are based upon SEM and TEM analysis of multiple samples from various zones of the wings of individuals, and of a limited number of samples from the body segments and from coprolites.

Type A scales are cover scales and are the most common scale type preserved on the wing. They are discussed in detail in the main text. Additional features of note include the absence of windows where scales taper basally (Figure S3a) (this is common in extant lepidopterans^18^) and the preservation of ultrastructural detail in brown, non-metallic, Type A scales (Figure S3b). Measurements of ultrastructural features in these scales are given in Table S2.

Type B scales are also cover scales but are rare, occurring only in basal and discal zones of the wing. They differ from Type A scales only in the structure of the lower part of the lumen: trabeculae are absent and the multilayer reflector is instead underlain by a 200-400 nm thick granular layer (Figure S3c, d).

Type C scales are rare and occur close to the inner margin of the forewing. These cover scales lack windows in the surficial layer, and microribs (typical spacing 170 nm) extend between adjacent ridges over the entire surface of the scale (Figure S3f, g); the lumen exhibits a granular texture (Figure S3e). The surface sculpture of these scales is identical to that seen in ‘satin-type’^16^ scales in extant lepidopterans.

Type D scales are ground scales and, as in extant lepidopterans, are usually obscured by the cover scales. They have been observed underneath only Type A and Type B scales; their occurrence underneath Type C scales cannot be confirmed. Their surface ornamentation is less pronounced than the associated Type A and Type B scales (Figure S3e). As in extant lepidopterans^16^, the multilayer structure is only weakly developed in contrast to the cover scales (Figure S3c, d).

Only Type A scales have been identified in coprolites (Figure S3h) and from the body segments; the absence of other scale types may reflect limited sampling. Scales in coprolites are often extensively wrinkled or convoluted, presumably a result of passage through an intestinal tract.

The wing membrane is preserved in individuals and exhibits a distinctive wrinkled texture similar to that in extant insects^18^ (Figure S3i).

Schematic reconstructions of the various scale types preserved in the fossil lepidopterans are shown in Figure S4.

**Supplementary References (otherwise numbers refer to references in the main text)**

50. Schulz, R., Harms, F.-J., & Felder, M. Die Forschungsbohrung Messel 2001: Ein Beitrag zur Entschlüsselung der Genese einer Ölschieferlagerstätte. *Z. Angew. Geol.* **4**, 9-17 (2002).

51. Felder, M. & Harms, F.-J. Lithologie und genetische Interpretation der vulkano-sedimentären Ablagerungen aus der Grube Messel anhand der Forschungsbohrung Messel 2001 und weiterer Bohrungen. *Cour. Forschungsinst. Senckenb.* **252**, 151-203 (2004).

52. Mertz, D.F. & Renne, P.R. A numerical age for the Messel fossil deposit (UNESCO World Heritage Site) derived from 40Ar/39Ar dating on a basaltic rock fragment. *Cour. Forschungsinst. Senckenb.* **255**, 67-75 (2005).

53. Kristensen, N.P., Scoble, M.J. & Karsholt, O. Lepidoptera phylogeny and systematics: the state of inventorying moth and butterfly diversity. *Zootaxa* **1668**, 1-76 (2007).

54. Tarmann, G. Generische Revision der amerikanischen Zygaenidae mit Beschreibung neuer Gattungen und Arten (Insecta:Lepidoptera). Teil II: Abbildungen. *Entomofauna*, **2**, Suppl. 2, 1-153 (1984).

55. Tarmann, G.M. Zygaenid moths of Australia: revision of the Zygaenidae of Australia (Procidinae: Artonini). 320 pp. (CSIRO, 2004).
